# Cellular and Molecular Targets in Acute Ischemic Stroke

**DOI:** 10.3390/ijms231911097

**Published:** 2022-09-21

**Authors:** Peter Kraft, Michael K. Schuhmann

**Affiliations:** 1 Department of Neurology, Klinikum Main-Spessart Lohr, Grafen-von-Rieneck-Str. 5, 97816 Lohr, Germany; 2 Department of Neurology, University Hospital Würzburg, Josef-Schneider-Str. 11, 97080 Würzburg, Germany

Despite the available treatment strategies, ischemic stroke (IS) is still a leading cause of death and disability worldwide. Therefore, novel cellular and molecular targets for potential clinical treatment applications in the future need to be identified. Until two decades ago, IS was mainly understood as the prototype of a mere thrombotic disease; however, nowadays there is clear evidence that IS rather involves the whole neurovascular unit (NVU) and interactions with immune cells and platelets. Innovation in this area of research has led to remarkable discoveries and improved understanding of the pathophysiology of IS. Nevertheless, more work and a tenacious pursuit of basic and clinical sciences in cerebrovascular pathophysiology and pharmacology are still necessary. The aim of this special issue is to present promising findings derived from both animal models and clinical studies of “Cellular and Molecular Targets in Acute Ischemic Stroke”, which might provide potential treatment strategies for future use.

A special focus was laid on the role of local vascular responses in acute stroke. The following studies paradigmatically delineate a crucial role of immune cell subsets and associated molecules/proteins on arterial thrombus formation [1], intravascular inflammation in hyper-acute stroke [2], ischemic affection/impairment of the NVU [3,4], and thus the blood-brain barrier (BBB) [5], as well as the direct translational approach of this special issue. Essig and co-workers characterized cerebral thromboemboli retrieved via endovascular thrombectomy regarding high-mobility group box 1 protein (HMGB1), a damage-associated molecular pattern (DAMP) involved in neutrophil extracellular trap (NET) formation and thrombosis. HMGB1 correlated to the amount of neutrophils and platelets. Co-localization of HMGB1 and NETs supports the notion that HMGB1 derived from platelets and NETosed neutrophils is a mediator in thrombus growth and cerebral thromboembolism. Therefore, despite platelet aggregation, platelet-driven inflammation might also be involved in arterial thrombus formation [1]. The same group analyzed samples of ischemic arterial blood aspirated directly from the pial cerebral collateral circulation during occlusive ischemia in patients with large vessel occlusion. They unraveled that leukocytes invade the occluded territory via retrograde collaterals during the hyper-acute phase of IS. This might lead to deterioration of collateral hemodynamics and infarct growth. The number of leukocytes (especially neutrophils) within the occluded cerebral vasculature was significantly higher compared to that in the systemic circulation and was associated with infarct extent [2]. Michalski et al., provide the first spatio-temporal characterization of the surfactant protein-G (SP-G) at the NVU after experimental focal cerebral ischemia in mice, rats, and sheep [3]. This study may stimulate further discussion about the role of SP-G within the ischemia affected NVU, which might be linked to the integrity of the vasculature. Endothelial cells represent the vascular interface for detrimental immune cell and platelet responses in the context of ischemia / reperfusion (I/R) injury. A study by Haarmann et al., analyzed the role of brain endothelial endoglin by characterizing its cellular expression, as well as the release of soluble endoglin (sENG) during hypoxia and subsequent reoxygenation. Data indicate that sENG functions as inflammatory mediator at the endothelium in the context of reperfusion injury in cerebral stroke [4]. Bieber et al., recently found that treatment with edoxaban attenuates I/R injury in mice by reducing BBB damage and inflammation [5], but further investigations are warranted.

Several other studies focused on potential therapeutic options to reduce ischemia induced neuronal cell death. Hong et al. investigated the role of temperature on apoptosis-mediated neuronal cell death in a rodent model of transient global ischemia, mimicking sudden cardiac arrest in humans. They found equal neuroprotective effects of targeted temperature management at 33 °C and 36 °C [6]. Another study carved out that atorvastatin has neuroprotective effects on global cerebral ischemia and helps to recover cognitive function [7]. Specific pathophysiological aspects of the putative relationship between low neuronal activity and apoptosis [8] and the regulatory role of H19 / miR-181a / ATG5 signaling in perinatal nicotine exposure-induced development of neonatal brain hypoxic-ischemic sensitive phenotype [9] are described in this special issue.

Furthermore, cellular and molecular targets for non-invasive, non-pharmacological therapeutic/rehabilitative interventions in acute IS [10] and the physiological and molecular pathways inherent to tolerant species that have been described to contribute to hypoxia tolerance [11] is systematically reviewed.

In summary, this special issue offers an interesting background and further insights of IS pathophysiology from both animal and clinical studies, and suggests novel strategies that may help in the ongoing efforts for a better prevention and treatment of cerebral ischemia.
